# Photodynamic antimicrobial chemotherapy in mice with *Pseudomonas aeruginosa*-infected wounds

**DOI:** 10.1371/journal.pone.0237851

**Published:** 2020-09-02

**Authors:** Zhan-Juan Zhao, Zeng-Ping Xu, Ying-Ying Ma, Jin-Duo Ma, Ge Hong

**Affiliations:** 1 School of Basic Medical Science, Hebei University, Baoding, China; 2 Institute of Biomedical Engineering, Chinese Academy of Medical Sciences & Peking Union Medical College, Tianjin Key Laboratory of Biomedical Material, Tianjin, China; Universidade de Aveiro, PORTUGAL

## Abstract

This study examined the antibacterial effect of protoporphyrin IX–ethylenediamine derivative (PPIX-ED)–mediated photodynamic antimicrobial chemotherapy (PPIX-ED-PACT) against *Pseudomonas aeruginosa in vitro* and *in vivo*. PPIX-ED potently inhibited the growth of *Pseudomonas aeruginosa* by inducing reactive oxygen species production via photoactivation. Atomic force microscopy revealed that PPIX-ED-PACT induced the leakage of bacterial content by degrading the bacterial membrane and wall. As revealed using acridine orange/ethidium bromide staining, PPIX-ED-PACT altered the permeability of the bacterial membrane. In addition, the antibacterial effect of PPIX-ED-PACT was demonstrated in an *in vivo* model of *P*. *aeruginosa*-infected wounds. PPIX-ED (100 μM) decreased the number of *P*. *aeruginosa* colony-forming units by 4.2 log_10_. Moreover, histological analysis illustrated that the wound healing rate was 98% on day 14 after treatment, which was 10% higher than that in the control group. According to the present findings, PPIX-ED-PACT can effectively inhibit the growth of *P*. *aeruginosa in vitro* and *in vivo*.

## Introduction

The emergence of multidrug-resistant bacteria (e.g., *Pseudomonas aeruginosa*) poses a major challenge to healthcare. *P*. *aeruginosa* is a common cause of nosocomial infections, especially in the intensive care unit [1] and operating room [2], as well as in prosthetic joints [3]. The pathogen is capable of producing several extracellular virulence factors that can cause extensive tissue damage, bloodstream invasion, and bacterial dissemination [4]. However, it is difficult to eradicate because of its natural resistance to existing antibiotics, thereby permitting the bacterium to easily cause sepsis and even death [5, 6]. Thus, the development of effective antimicrobials, especially those targeting infections associated with *P*. *aeruginosa*, is highly required.

Photodynamic antimicrobial chemotherapy (PACT), also known as antimicrobial photodynamic therapy, has aroused attention as an innovative and alternative treatment option. PACT, a potential antimicrobial therapy that uses visible light and a photosensitizer, can kill cells by inducing the production of reactive oxygen species (ROS) such as singlet oxygen (^1^O_2_) [7–11]. Its application has aroused interest in the field of antimicrobial chemotherapy because of its unique advantages, especially the low possibility of resistance among microbes [12–14]. ROS can cause cell death [15] by inducing DNA [16] and cell membrane damage [17], protein carbonylation [18], and lipid peroxidation [19].

The photodynamic effects of different photosensitizers on *P*. *aeruginosa* have been demonstrated [20–23]. *In vivo*, PACT has been primarily used to improve wound healing. The types of wounds treated with PACT have included burns on the dorsal skin of Wistar mice [24, 25]; abrasions on the dorsal skin of BALB/c [26, 27] and Wistar albino mice [28]; and excisional wounds in Wistar [29–31], Swiss albino [32, 33], hairless SKH-1 [34], C57BL/ksj db/db [35], and albino mice [36]. However, only two studies have assessed the use of PACT for treating *P*. *aeruginosa* infection [32, 33].

The photosensitizer is a crucial element of PACT, and porphyrins are capable of efficiently killing gram-positive bacteria [37], inhibiting the secretion of inflammatory factors, and promoting wound healing after burn wound infection [38]. Meantime, cationic and noncationic photosensitizers can ablate gram-negative bacteria in combination with agents that permeabilize the highly organized outer membrane of the microbes [39, 40]. Jori *et al*. [41] reported that a targeted polycationic photosensitizer can effectively eliminate methicillin-resistant *Staphylococcus aureus* (MRSA) and *Escherichia coli* from epidermal wounds and burns. The efficacy of PACT is associated with the uptake of the photosensitizer by the target bacteria [42], and the beneficial effects are independent of the antibiotic resistance pattern in microbial strains [41]. An ideal photosensitizer exhibiting high biocompatibility, easy absorption, and suitable photophysical parameters for PACT can confer potent antibacterial effects. Moreover, the optimal photosensitizer should display high biocompatibility, high stability, and high affinity for bacterial cells, thereby increasing its uptake and antibacterial activity. Polyamine, a substance required for cellular development, can increase biocompatibility and activity when conjugated to a Protoporphyrin IX (PPIX) [43]. Accordingly, this study hypothesized that PPIX conjugates can alter the permeability of the bacterial outer membrane and exert significant phototoxic effects against gram-positive bacteria.

**Fig 1:**
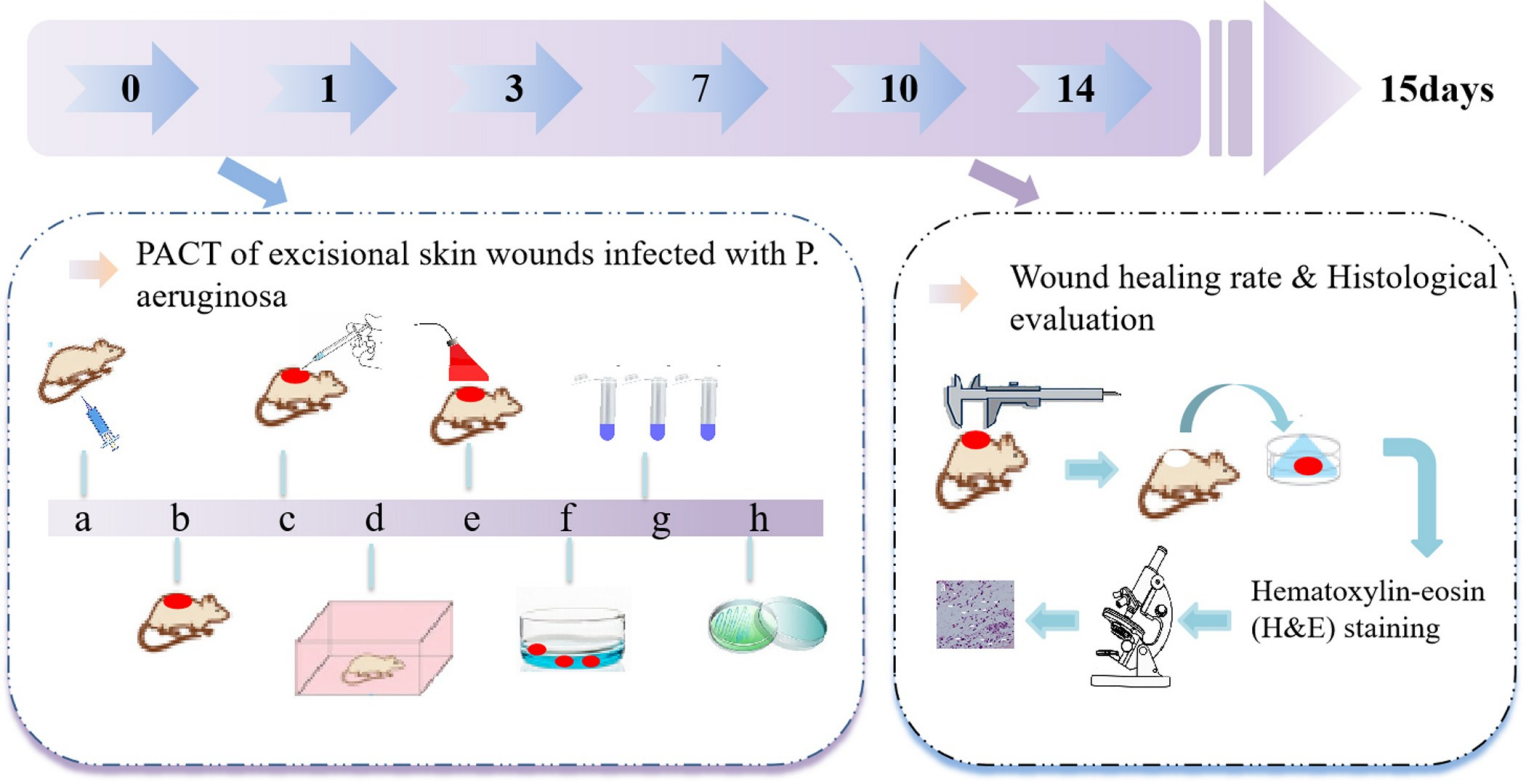
Model of wound infection treated with photodynamic antimicrobial chemotherapy and the detection procedure.

In this study, a photosensitizer was designed by conjugating PPIX with an ethylenediamine derivative (termed PPIX-ED), and the antibacterial effect of the photosensitizer in excisional wounds infected with *P*. *aeruginosa* was assessed (Fig 1).

## Materials and methods

### Chemicals

**Fig 2:**
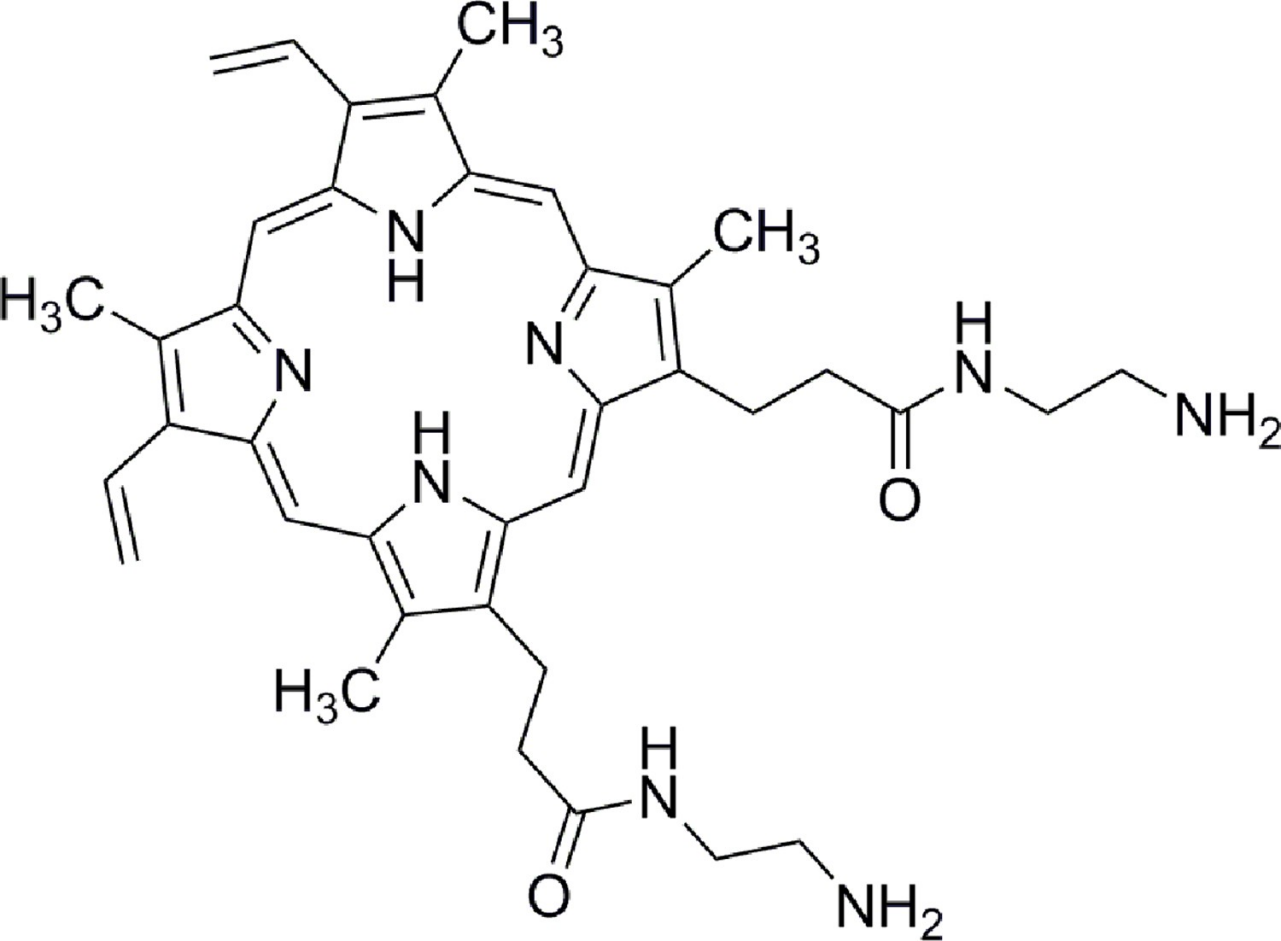
Structure of *N*, *N*′-bis (2-aminoethyl) -2,7,12,18-tetramethyl-3,8-divinyl-21*H*, 23*H*-porphyrin-13,17-bispropanamide porphyrin (PPIX-ED).

In the supporting information, we described the synthesis and characterization of PPIX-ED. The chemical structure of PPIX-ED is presented in Fig 2. The chemical name is *N*, *N'*-bis (2-aminoethyl)-2,7,12,18-tetramethyl-3,8-divinyl-21*H*, 23*H*-porphyrin-13,17-bispropanamide. A stock solution (500 μM) was prepared by dissolving the target compounds in DMSO and subsequently stored at −20°C in the dark before use.

### Light source

A semiconductor laser (Model 7404, Intense Inc., North Brunswick, NJ, USA) with a wavelength of 650 nm was used in this experiment, and the energy density was determined using an optical power meter (LM1; Carl Zeiss).

### Photobleaching

The photobleaching experiment was conducted using a multimode microplate spectrophotometer [44]. Briefly, dye and buffer solutions were prepared immediately before measurements. A 200 μL sample of 2 × 10^−5^ M dye in 96-well microtiter plates was sealed with cover slips to avoid evaporation. Photobleaching measurements at 300–800 nm were conducted for 30 min, and data were recorded every 5 min. The delivered light energy was nearly 0.2 J/cm^2^ per min, and the total light energy density was 6 J/cm^2^.

### Measurement of the quantum yield of ^1^O_2_ (Φ_Δ_)

Φ_Δ_ was determined by the decomposition of 1,3-diphenyl isobenzofuran (DPBF) in DMSO, and Φ_Δ_ is correlated with the decay of the absorption of light at 410 nm by DPBF [45]. The sample was dissolved in DMSO and irradiated at 650 nm (5 mW/cm^2^). The following Eq (1) was used to calculate the rate of ^1^O_2_ generation using 5,10,15,20-tetraphenylporphyrin (TPP) as the reference ($\Phi_{\Delta }^{R}$ = 0.64) [46].

$$\Phi_{\Delta }^{S}=\Phi_{\Delta }^{R}\frac{{\text{K}}^{\text{S}}{\text{F}}^{\text{R}}}{{\text{K}}^{\text{R}}{\text{F}}^{\text{S}}},$$ (1)

where $\Phi_{\Delta }^{R}$ is the quantum yield of ^1^O_2_ for the reference; superscript S and R indicate the sample and reference compound, respectively; K is the slope of the plot of the change in absorbance by DPBF (at 410 nm) with prolonged irradiation; F is the absorption correction factor, which is given by F = 1 – 10^−OD^; and OD is the corresponding absorbance at the radiation wavelength.

### Bacterial culture

The *P*. *aeruginosa* strain used in this study (ATCC-27853) was donated by the First Affiliated (304) Hospital, Chinese PLA General Hospital. Multiple single colonies were picked and inoculated in 10 mL of Luria–Bertani (LB) medium. The medium was cultured on a shaker (200 rpm) at 37°C, and its optical density (OD) was determined at 600 nm (OD_600_). The bacteria were collected via centrifugation when OD_600_ reached nearly 0.7 and resuspended in an equal volume of PBS [47].

### Fibroblast culture

NIH3T3 (SCSP-515) mouse fibroblasts [48, 49], as an example of normal cells, were purchased from the Cell Bank of the Chinese Academy of Sciences (Shanghai, China) and used to evaluate the toxicity of PPIX-ED. The cells were cultured in Dulbecco’s modified Eagle’s medium (Sigma-Aldrich, UK) containing 10% heat-inactivated fetal bovine serum (Gibco), penicillin (100 units/mL), and streptomycin (100 g/mL) (Sigma-Aldrich) and incubated at 37°C in a humidified atmosphere of 5% CO_2_ until the cell monolayer reached at least 80% confluence. Cells were then washed with PBS, incubated for 3 min at 37°C with 0.05% trypsin and 0.02% EDTA, seeded into 96-well cell culture plates (8 × 10^3^–1 × 10^4^ cells/well), and incubated overnight to permit reattachment. All subsequent photoinactivation experiments were performed in 96-well cell culture plates.

### *In vitro* PACT studies

#### Bacteria survival assay

The number of bacterial colonies was described as colony-forming units (CFUs). Mixtures of the bacterial suspensions (1 × 10^7^ CFU/mL) and different concentrations of the photosensitizer (3.13, 6.25, 12.5, 25.0, and 50.0 μM) were added to a 96-well plate and incubated in the dark at 37°C for 30 min. Then, the mixture was irradiated with a laser at 6 J/cm^2^. Next, 100 μL of the mixture were taken from each well, and each gradient dilution of the mixtures (1 × 10^−1^, 1 × 10^−2^, 1 × 10^−3^, 1 × 10^−4^, 1 × 10^−5^) was spread on LB agar plates and incubated at 37°C for 18 h in the dark. Eventually, the CFUs were counted. The experiment was repeated three times. Bacterial survival was expressed as a ratio of the number of CFUs of bacteria treated with light and the photosensitizer to that of untreated bacteria [50].

#### Cell survival assay

After 24 h of growth at 37°C in 5% CO_2_, the cells were then incubated with PPIX-ED at various concentrations (3.13, 6.25, 12.5, 25.0, and 50.0 μM) at 37°C for 30 min in the dark, irradiated with 6 J/cm^2^ light for 30 min, and incubated overnight at 37°C. Twenty-four hours after photodynamic therapy, 5 mg/mL (3-(4,5-dimethylthiazol-2-yl)2,5-diphenyl tetrazolium bromide (MTT, Thermo Fisher Scientific, USA) was added to the cells, which were incubated at 37°C to allow cleavage of the tetrazolium ring by mitochondrial dehydrogenases and the formation of blue formazan crystals in living cells. After 3 h, the supernatant was removed, and the crystals were dissolved in DMSO (Sigma-Aldrich). The absorption of the formazan in each well was determined at 490 nm using a microplate reader (Varioskan Flash Multimode Reader, Thermo Fisher Scientific). All assays were performed in the dark [51, 52].

#### Uptake study

The uptake experiments were performed as described by Soukos *et al*. [51]. In general, 1 × 10^3^ μL of bacterial suspensions were centrifuged (9000 × *g*, 1 min) and resuspended in PBS until OD_600_ = 0.7. PPIX-ED was added at a final concentration of 3.13–50.0 μM. The mixtures were incubated in the dark for 30 min at ambient temperature, centrifuged at 9000 × *g* for 1 min, and then washed with PBS to remove residual photosensitizer. The bacterial pellet was dissolved in 1 mL of a 10% aqueous solution of sodium lauryl sulfate and allowed to stand for 24 h to fully release the absorbed photosensitizer. The uptake of the photosensitizer by the bacteria was determined using a fluorescence assay. The fluorescence of PPIX-ED was measured (λex = 406 nm, λem = 604 nm) and normalized as previously described. The standard curve was obtained by plotting known concentrations of the target compounds against the fluorescence intensity. Uptake was calculated by comparing the determined fluorescence intensity with the standard curve at different time points. A blank control group without a photosensitizer was created.

#### Confocal laser-scanning microscopy

*P*. *aeruginosa* was suspended in PBS to an appropriate cell density (OD_600_ = 0.7) and treated with PPIX-ED at a final concentration of 12.5 μM for 30 min at ambient temperature. The bacteria were then harvested via centrifugation (9000 × *g*, 1 min) and washed twice with PBS, and one drop of this suspension was placed onto a confocal dish and dried at ambient temperature. Images of fluorescent bacteria were captured using a confocal laser-scanning microscope (LSM710; Carl Zeiss) at an excitation wavelength of 405 nm and emission wavelength of 650 nm.

Nearly 2 × 10^4^ NIH3T3 cells in culture medium (2 mL) were seeded on a confocal dish and incubated overnight at 37°C in 5% CO_2_. The medium was removed, and the cells were incubated with PPIX-ED (12.5 μM) for 30 min under the same conditions used for *P*. *aeruginosa*. Then, the cells were rinsed with PBS and characterized using an LSM 710 confocal microscope as described previously [42].

#### Fluorescence imaging

The samples were mixed with 4 μL of 100 mg/L acridine orange (AO)/100 mg/L ethidium bromide (EB) dual fluorescent dye and stained for 5 min in the dark to characterize bacterial membrane permeability after PPIX-ED-PACT. Five microliters of the mixture were taken and dropped on a glass slide. A fluorescence image was immediately taken using a fluorescence microscope (Nikon Eclipse Ti/B0004, Nikon, Japan). The image magnification is ×200 [38].

#### Atomic force microscopy (AFM) imaging

Bacterial samples (1 × 10^7^ CFU/mL) were dropped on the surface of the mica plate (approximately 0.5 cm^2^), air-dried at ambient temperature, and then scanned using an atomic force microscope (Veeco Multi Mode 8/B0021, Veeco German) equipped with an antimony-doped conical silicon (Veeco) in an intelligent mode [22]. The force constant was 3 N/m.

### *In vivo* PPIX-ED-PACT studies

#### PACT of excisional skin wounds infected with *P*. *aeruginosa*

Female BALB/c mice (6–8 weeks old, 17–22 g, Beijing Huafukang Biotechnology Co., Ltd, China) were maintained under standard nutrition and housing conditions. The breeding room was specific pathogen-free, and the breeding temperature was 20–25°C. The BALB/c mice were fed a full-price nutritious pellet feed supplemented with a certain proportion of crude fiber, and the feed and drinking water were given regularly and quantitatively on a day-to-day basis. The present study, abiding by institutional guidelines, was approved by the Laboratory Animal Management Committee/Laboratory Animal Welfare Ethics Committee, Institute of Radiation Medicine, Chinese Academy of Medical Sciences.

Before skin preparation, mice were anesthetized with intraperitoneal injections of 1% sodium pentobarbital (45 mg/kg) until their respiratory rates decreased, the depth of respiration increased, the eyelid and corneal reflexes disappeared, the muscle tension and reflex response decreased, and no response to pain was generated. The back of each animal was shaved using an electric razor and then treated with a depilatory agent. Excisional wounds were created by pinching and lifting the skin of the back with sterile forceps and cutting a 10-mm circular (78.5 mm^2^) area with scissors. Five minutes after wounding, 50 μL of a suspension supplemented with 1 × 10^9^ CFUs of *P*. *aeruginosa* in PBS were added, followed by incubation for 30 min. Subsequently, 100 μL of PPIX-ED (50, 100, or 200 μM) were added, followed by incubation for an additional 30 min. Next, the wounds were irradiated with a 650-nm laser (60 J/cm^2^) for 10 min. Bandages were wrapped around the wound of each mouse to protect the shaved skin and wounds from other harm. All mice were sacrificed via cervical dislocation on day 15 after treatment.

#### Bacterial counts in the wound after PACT

When the treatment was completed, a circular area of skin and subcutaneous tissue (1 cm diameter) associated with the wound at the center were removed with sterile scissors, placed in 0.5 mL of sterile PBS, immediately minced to release the bacteria within the wound, and subsequently harvested in an Eppendorf tube containing 4.5 mL of PBS. Aliquots were serially diluted 10-fold, and each diluted sample was plated onto a LB plate and then incubated at 37°C in air for 36 h. The data are expressed as the mean CFUs of *P*. *aeruginosa* recovered from each wound.

#### Wound healing rate

The widths and lengths of the wounds were determined using Vernier calipers on days 1, 3, 7, 10, and 14 after treatment.

#### Histological evaluation

The treated wounds were visually inspected daily, removed 7 and 14 days after treatment, and fixed in 10% formalin for 24 h. Specimens taken from the boundary of the wound and adjacent unaffected skin were processed and embedded in paraffin wax. Sections (6 μm thick) were stained with hematoxylin-eosin and examined via light microscopy.

### Statistical analysis

Statistical analysis was performed using SPSS 19.0. Data were analyzed using Student’s *t*-test and expressed as the mean ± standard error. *P* < 0.05 denoted statistical significance.

## Results

### Photostability and Φ_Δ_

**Fig 3:**
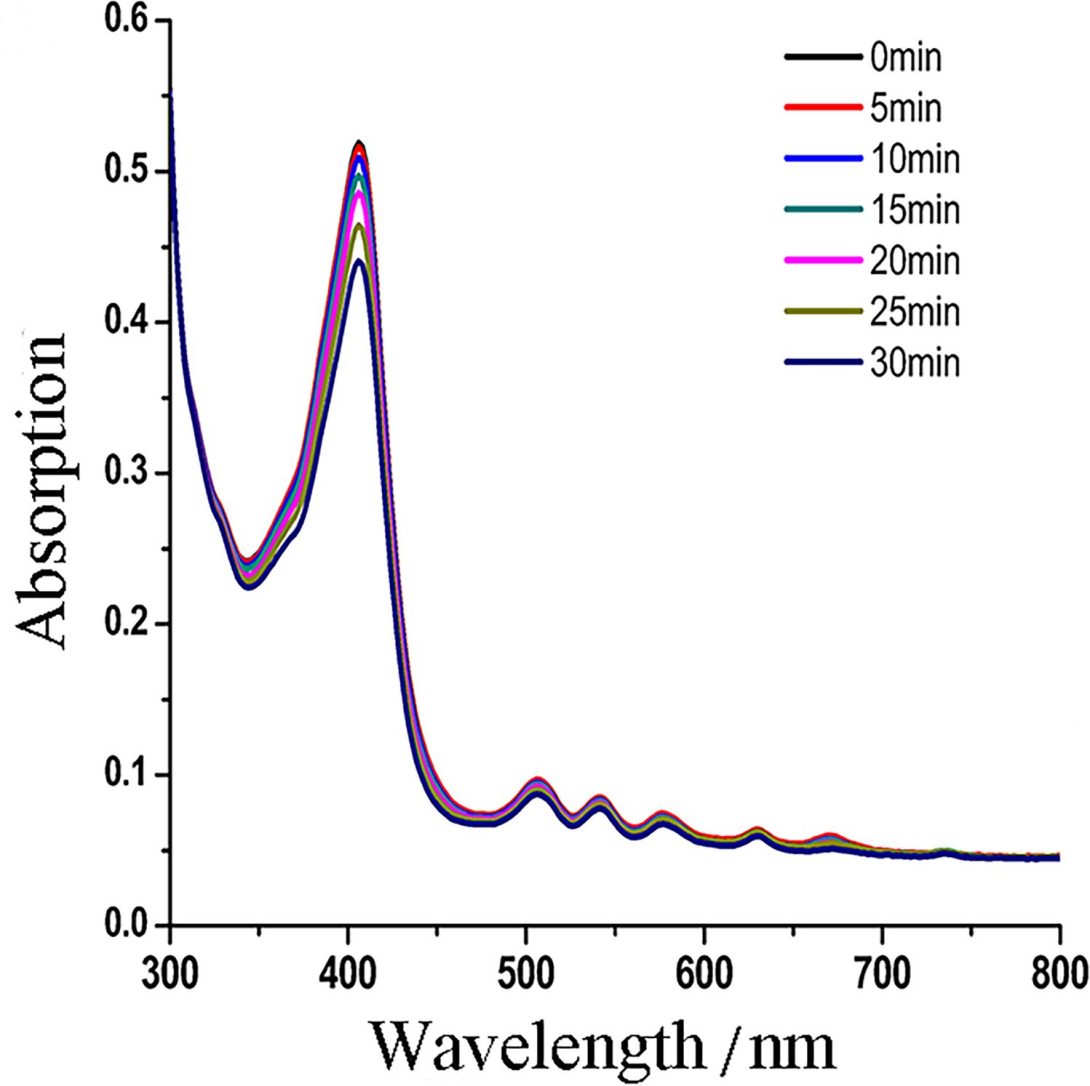
Photobleaching of protoporphyrin IX–ethylenediamine derivative (PPIX-ED). A photobleaching experiment was performed using a multimode microplate spectrophotometer. The photostability profiles of PPIX-ED under different irradiation times were obtained (2 × 10^−5^ mol/L PPIX-ED, 4.2 mW/cm^2^ light energy density). The absorption spectrum of PPIX-ED spanned 0–30 min.

We investigated the photostability of PPIX-ED by assessing photobleaching using a multimode microplate spectrophotometer at intervals of 5–30 min. Photobleaching experiments revealed that after irradiation with 650 nm light (6 J/cm^2^) for 30 min, the corresponding absorption spectra and related ODs of PPIX-ED changed by less than 15% (Fig 3). These data revealed that PPIX-ED has relatively high photostability.

An ideal photosensitizer should achieve high Φ_Δ_. In this study, DPBF acted as a reducing agent to trap singlet ^1^O_2_ using TPP as the reference. Φ_Δ_ of PPIX-ED reached 0.68 when a mixture of DPBF (2 × 10^−5^ mol/L) and PPIX-ED was irradiated with a 650-nm semiconductor laser (5 mW/cm^2^). The DPBF decay rate was higher in the presence of PPIX-ED, revealing it was more efficient than TPP in producing ^1^O_2_.

### Uptake of PPIX-ED

**Fig 4:**
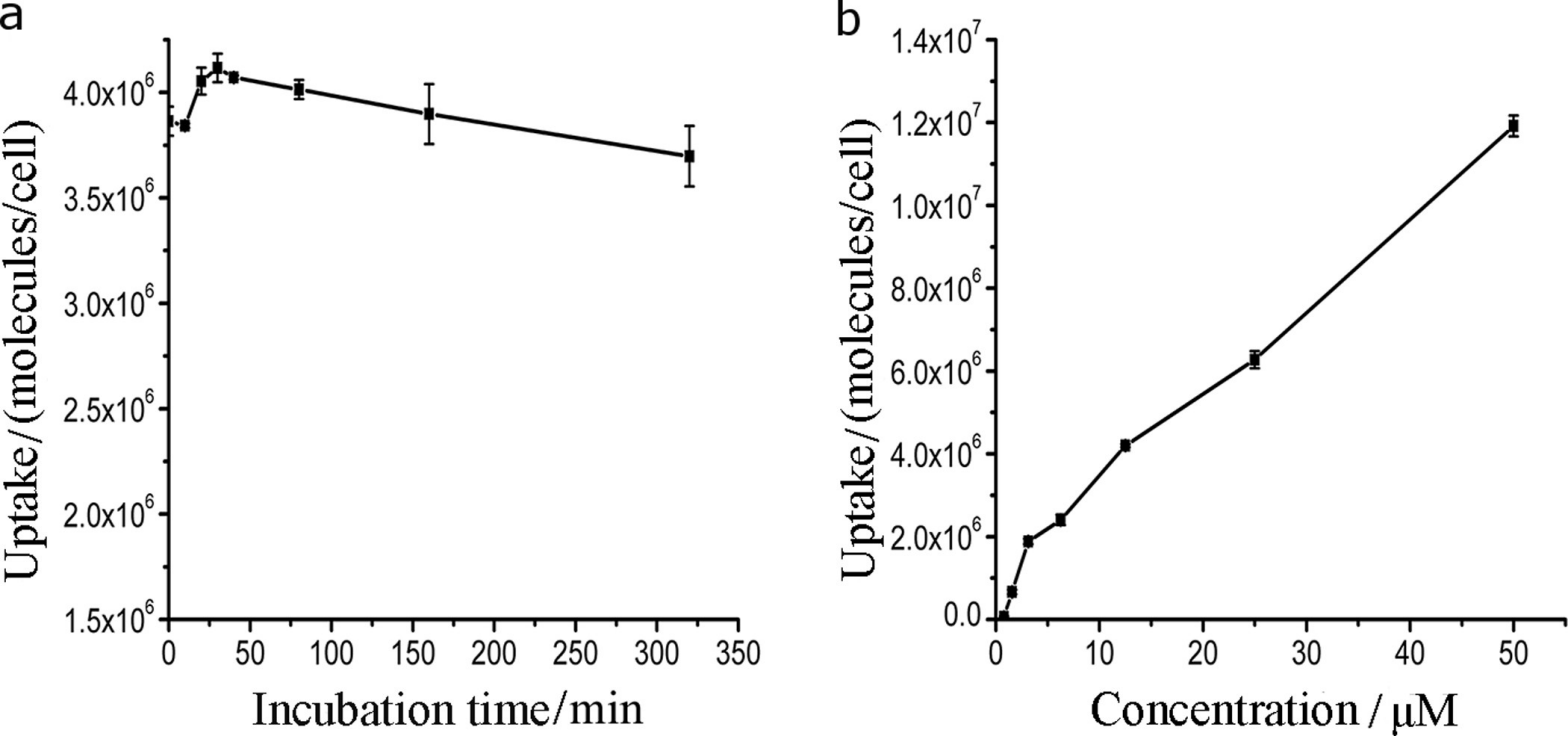
Uptake of protoporphyrin IX–ethylenediamine derivative (PPIX-ED) by *Pseudomonas aeruginosa* in relation to the incubation time and concentration. The uptake was evaluated using a fluorescence assay. (a) The bacterial culture was incubated with PPIX-ED (6.25 μM) for 320 min or with (b) PPIX-ED at a final concentration of 3.13–50.0 μM in the dark for 30 min at ambient temperature.

The bacterial culture was incubated with PPIX-ED (6.25 μM) for 320 min, and the absorption of the photosensitizer by the bacteria at different periods was determined spectrophotometrically [42]. The uptake of PPIX-ED by *P*. *aeruginosa* was concentration- and time-dependent (Fig 4). Fig 4A reveals that the maximal uptake of PPIX-ED by *P*. *aeruginosa* occurred after 30 min. The maximal uptake and efficacy of PACT were closely related to the incubation time and amount of the compound. Accordingly, the incubation time was set at 30 min for the photoreaction and dark control reactions in the subsequent *in vitro* and *in vivo* experiments.

### *In vitro* photoinactivation of bacteria mediated by PPIX-ED

**Fig 5:**
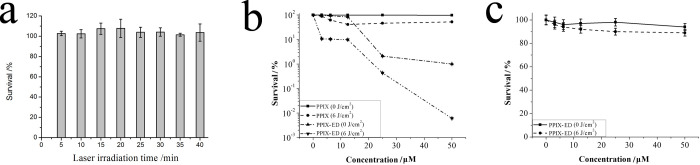
Effects of (a) light alone on *Pseudomonas aeruginosa* and (b) photoinactivation by protoporphyrin IX–ethylenediamine derivative (PPIX-ED) and PPIX in *P*. *aeruginosa*. (c) Photoinactivation of NIH3T3 cells by PPIX-ED. The bacteria and fibroblasts were incubated with the compounds over the concentration range of 3.13–50.0 μM for 30 min followed by exposure to 650 nm irradiation (6 J/cm^2^) or dark exposure. The results are expressed as the mean and standard deviation.

To assess the photoinactivation activity of PPIX-ED in *P*. *aeruginosa*, five treatment groups were created: light only, PPIX-ED only, PPIX-ED + light, PPIX only, and PPIX + light. *P*. *aeruginosa* suspensions were incubated with the compound in the dark for 30 min at 37°C. Fig 5 illustrates the photodynamic efficacy of the laser and photosensitizers in *P*. *aeruginosa*. There was no significant difference among the light (Fig 5A), PPIX-ED only (Fig 5B), and PPIX groups (Fig 5B). The bacterial survival rate increased slightly, which indicates that the laser or PPIX-ED alone had no or weak effects on the growth of *P*. *aeruginosa*. However, in the PPIX-ED-PACT group, the growth of the bacteria was substantially inhibited, with the inhibition rate exceeding 99% at 25 μM (Fig 5B). As a control, PPIX had weak bactericidal effects against *P*. *aeruginosa* under light or dark conditions. The results revealed that PPIX-ED exerted strong photoinactivating effects on *P*. *aeruginosa*.

#### Phototoxicity of PPIX-ED in NIH3T3 cells

NIH3T3 cells were used to assess the phototoxicity of PPIX-ED using the MTT assay. NIH3T3 cells were incubated with PPIX-ED under the identical conditions employed for *P*. *aeruginosa*. The phototoxicity of PPIX-ED in NIH3T3 cells was concentration-dependent (Fig 5C). In dark conditions, the survival of NIH3T3 exceeded 95% over the concentration range of 0–50.0 μM. After irradiation with 6 J/cm^2^, NIH3T3 cell survival exceeded 88.5% at 25.0 μM, a concentration at which *P*. *aeruginosa* was almost fully eliminated. This suggested that PPIX-ED does not cause significant cellular damage at concentrations with high photoinactivation efficacy against *P*. *aeruginosa*, which indicates that it can be safely used in antibacterial strategies.

#### Confocal laser-scanning microscopy images of *P*. *aeruginosa* treated with photosensitizers

**Fig 6:**
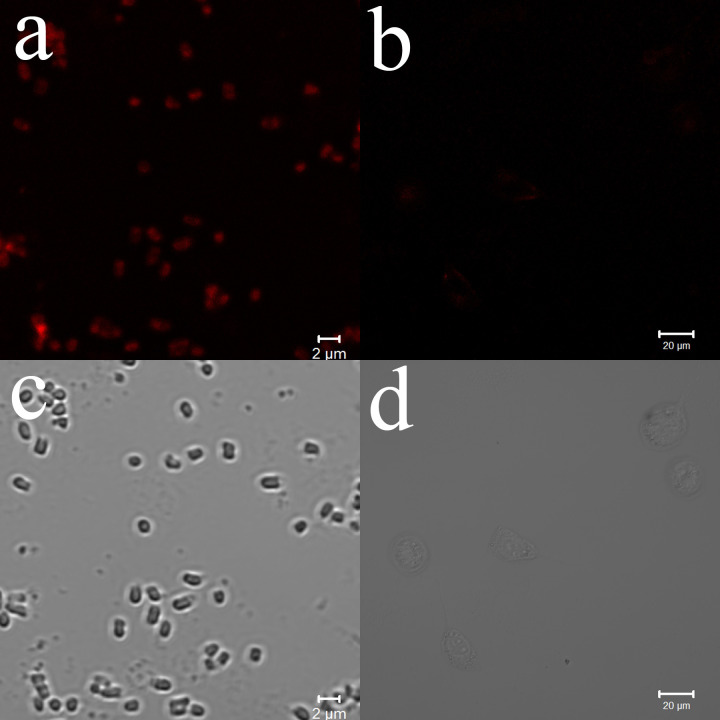
Confocal laser-scanning microscopy of (a) *Pseudomonas aeruginosa* and (b) NIH3T3 cells after incubation with 12.5 μM protoporphyrin IX–ethylenediamine derivative (PPIX-ED) for 30 min. (c) Optical image of untreated *P*. *aeruginosa*. (d) Optical image of untreated NIH3T3 cells.

Confocal laser-scanning microscopy was adopted to characterize the uptake of PPIX-ED by *P*. *aeruginosa*. It is easy to monitor the uptake of a photosensitizer by bacteria using a fluorescence microscopy system. Fig 6 indicates that PPIX-ED was accumulated by *P*. *aeruginosa* and NIH3T3 cells after 30 min of incubation. NIH3T3 cells incubated with PPIX-ED exhibited relatively weak fluorescence signals, whereas the intensity of the red fluorescence emitted from *P*. *aeruginosa* incubated with PPIX-ED was intense. This suggested that *P*. *aeruginosa* had higher uptake of PPIX-ED than NIH3T3 cells, which could explain the weak phototoxicity of PPIX-ED in NIH3T3 cells.

#### Membrane integrity

**Fig 7:**
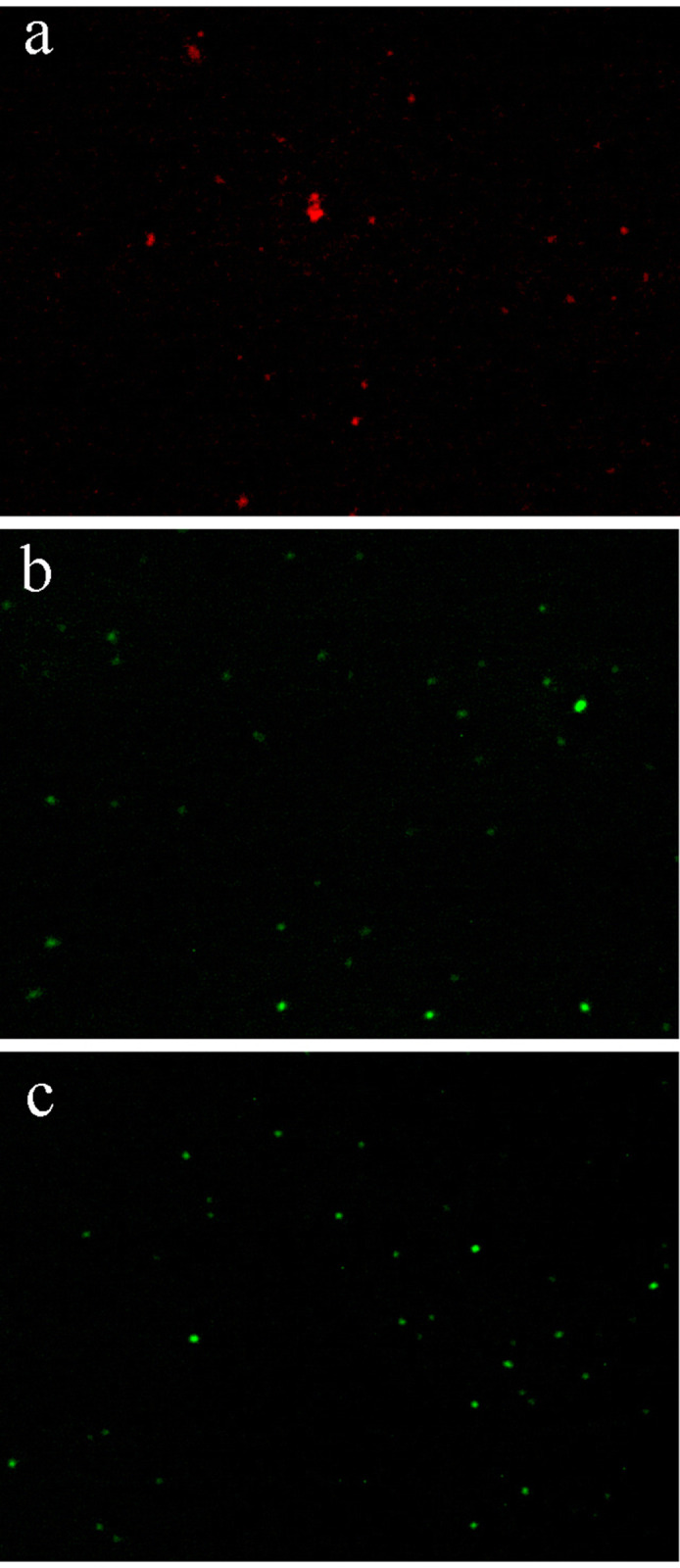
Fluorescence microscopy images of *Pseudomonas aeruginosa*. The *P*. *aeruginosa* were exposed to (a) protoporphyrin IX–ethylenediamine derivative (PPIX-ED)–mediated photodynamic antimicrobial chemotherapy (PACT), (b) PPIX-ED with no light (b), or (c) no treatment (control group).

The permeability of the bacterial membrane before and after PPIX-ED-PACT was measured using fluorescence microscopy. The antibacterial activity of PPIX-ED-PACT was assessed using a dual fluorescent dye (AO/EB) as an indicator of bacterial membrane damage. Living *P*. *aeruginosa* bacteria emitted green fluorescence, and dead bacteria emitted red fluorescence. Fig 7C presents the fluorescence microscopy images of the control samples that were not exposed to PPIX-ED. The bacteria were dispersed and alive, as indicated by their green fluorescence. As presented in Fig 7A, most of the dead bacteria emitted red fluorescence in the PPIX-ED–PACT group. Moreover, the fluorescence microscopy images of *P*. *aeruginosa* treated with 12.5 μM PPIX-ED without illumination revealed weak bactericidal effects (Fig 7B).

The aforementioned results indicated that PPIX-ED-PACT can damage the bacterial membrane and alter its permeability.

#### Morphological changes

**Fig 8:**
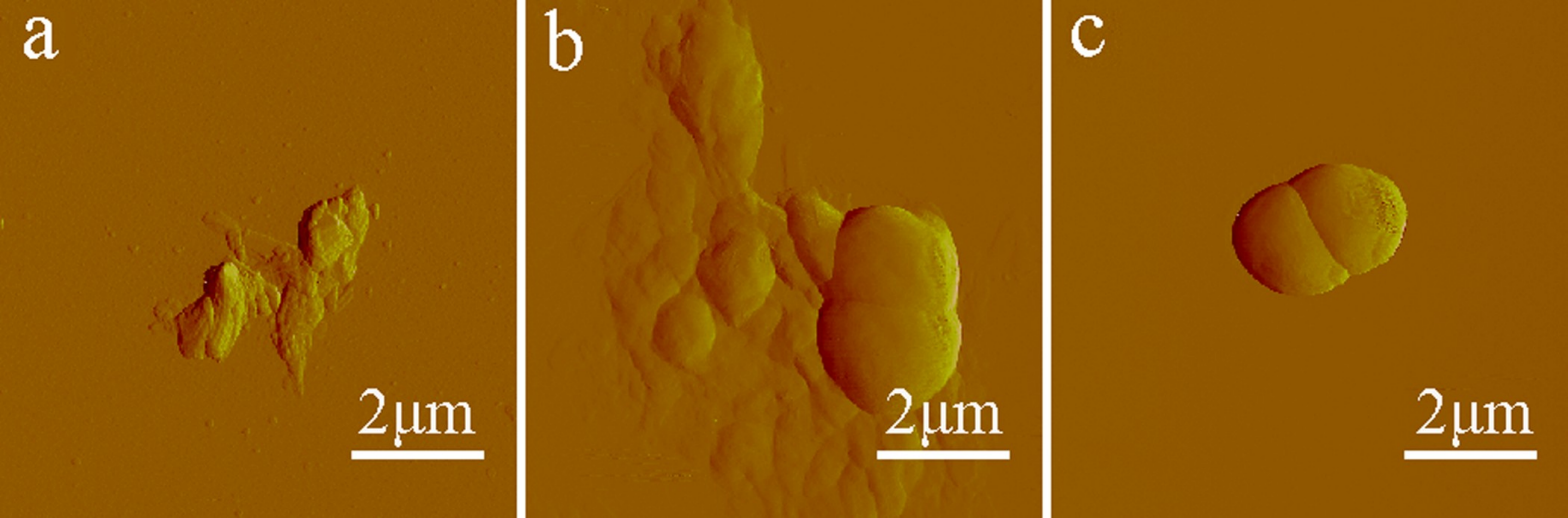
Atomic force microscopy images of *Pseudomonas aeruginosa*. *P*. *aeruginosa* was exposed to (a) protoporphyrin IX–ethylenediamine derivative (PPIX-ED)–mediated photodynamic antimicrobial chemotherapy (PACT), (b) PPIX-ED with no light (b), or (c) no treatment (control group).

Variations of the bacterial structure could be clearly identified under an atomic force microscope at the morphological level. Bacterial samples (1 × 10^7^ CFU/mL) were dropped onto the surface of the mica plate, dried at ambient temperature naturally, and subsequently scanned in an intelligent mode [53]. In the PPIX-ED-PACT group (Fig 8A), *P*. *aeruginosa* was fully distorted and broken into pieces. Some irregular aggregation of the dead bacteria was detected. However, the rod-like shape of *P*. *aeruginosa* was intact in the control group (Fig 8C). In the group treated with PPIX-ED in the dark (Fig 8B), the bacterial structure featured rough surfaces and blurred edges. Conversely, the dimensions remained almost unchanged, and the particles scattered on the surface of *P*. *aeruginosa* might have been the aggregates of PPIX-ED. Thus, PPIX-ED-PACT exerted potent effects on the bacterial envelope, including damage to the bacterial wall and membrane.

### *In vivo* antibacterial activity

**Fig 9:**
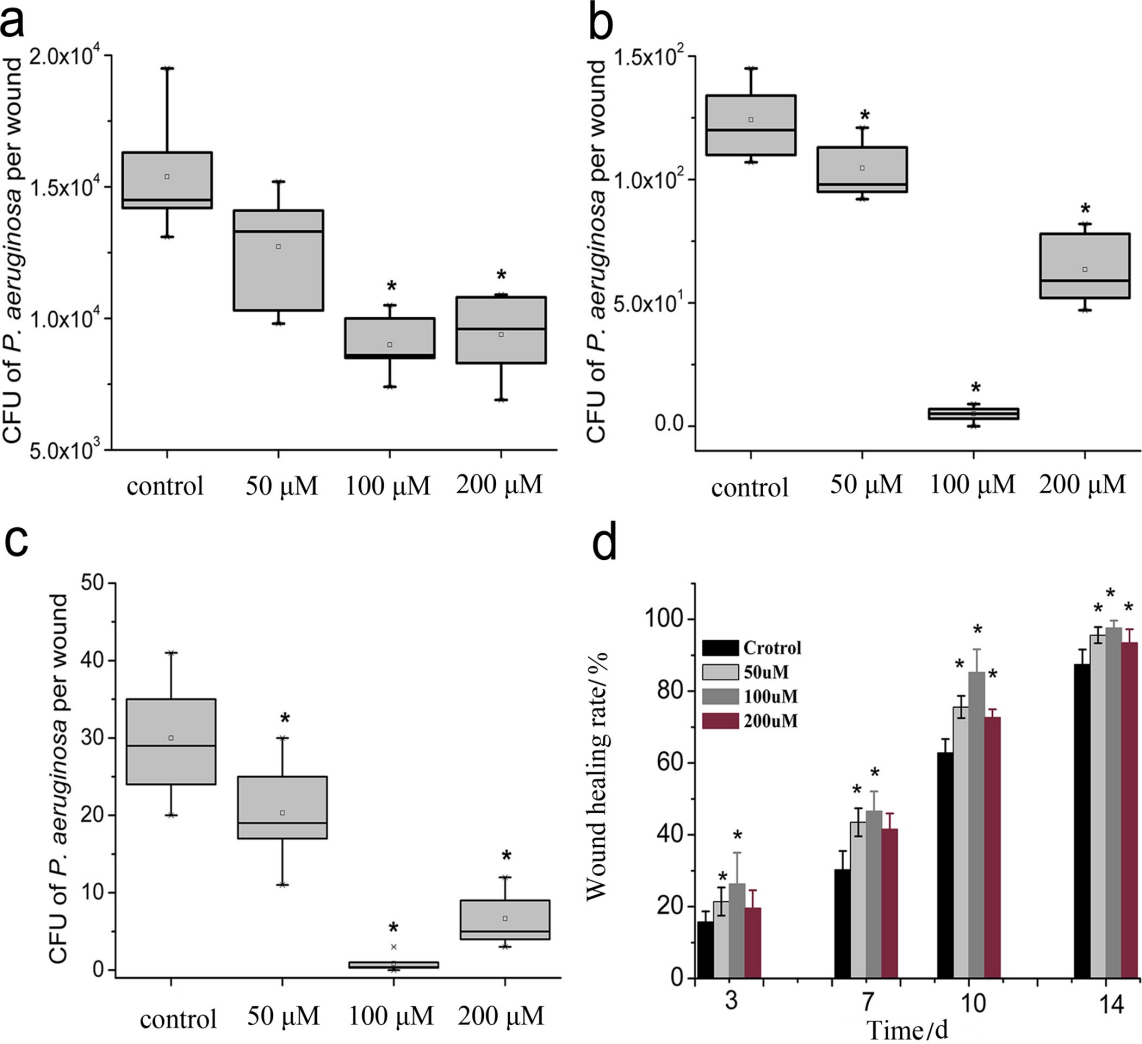
Box plot of the viability of bacteria in wounded tissue in mice on days (a) 1, (b) 10, and (c) 14 (*P < 0.05, significant difference versus the control group). (d) Wound healing rate in the protoporphyrin IX–ethylenediamine derivative-mediated photodynamic antimicrobial chemotherapy (PPIX-ED-PACT) and control groups on days 3, 7, 10, and 14 after treatment (*P < 0.05, significant difference versus the control group).

Based on the preliminary experiments, the *in vivo* antibacterial effect of PPIX-ED-PACT was analyzed at different PPIX-ED concentrations (50, 100, and 200 μM). The viable counts of *P*. *aeruginosa* in wounds were assessed *in vivo* to clarify the antibacterial effect of PPIX-ED-PACT (Fig 9A–9C). The control group (wounds without any treatment) exhibited greater bacterial viability at each time point. On days 1, 10, and 14 after treatment, the PACT-treated groups displayed obvious reductions in viable bacteria counts compared with those in the control group. At identical time points, the decrease in the viable bacteria count was related to the PPIX-ED concentration as follows: 100 μM > 200 μM > 50 μM. On day 14 after treatment, the CFUs of *P*. *aeruginosa* were decreased by factors of 4.2, 3.2, and 3.0 log_10_ in the 100, 50, and 200 μM groups, respectively, compared with 2.6 log_10_ in the control group.

#### Wound healing rate

We compared the wound healing rate over time between the control and various PPIX-ED groups (50, 100, 200 μM) after PACT. The wound healing rates of the PACT-treated and control groups (n = 10 each) are presented in Fig 9D. Statistical analysis revealed that the wound healing rate was higher in the PACT-treated groups than in the control group, and there were significant difference on days 3, 7, and 10 but not day 14. Moreover, the wound healing rate was highest in the 100 μM PPIX-ED-PACT group. On day 14 after treatment, the average wound healing rates reached 97.6 ± 2.0%, 95.6 ± 2.3%, and 93.5 ± 3.8% in the 100, 50, and 200 μM PPIX-ED-PACT groups, respectively, compared with 87.9 ± 3.9% in the control group. Nearly total wound closure was identified in the 100 μM PPIX-ED-PACT group, whereas the wound in the control group was unclosed.

#### Histological analysis following PACT

**Fig 10:**
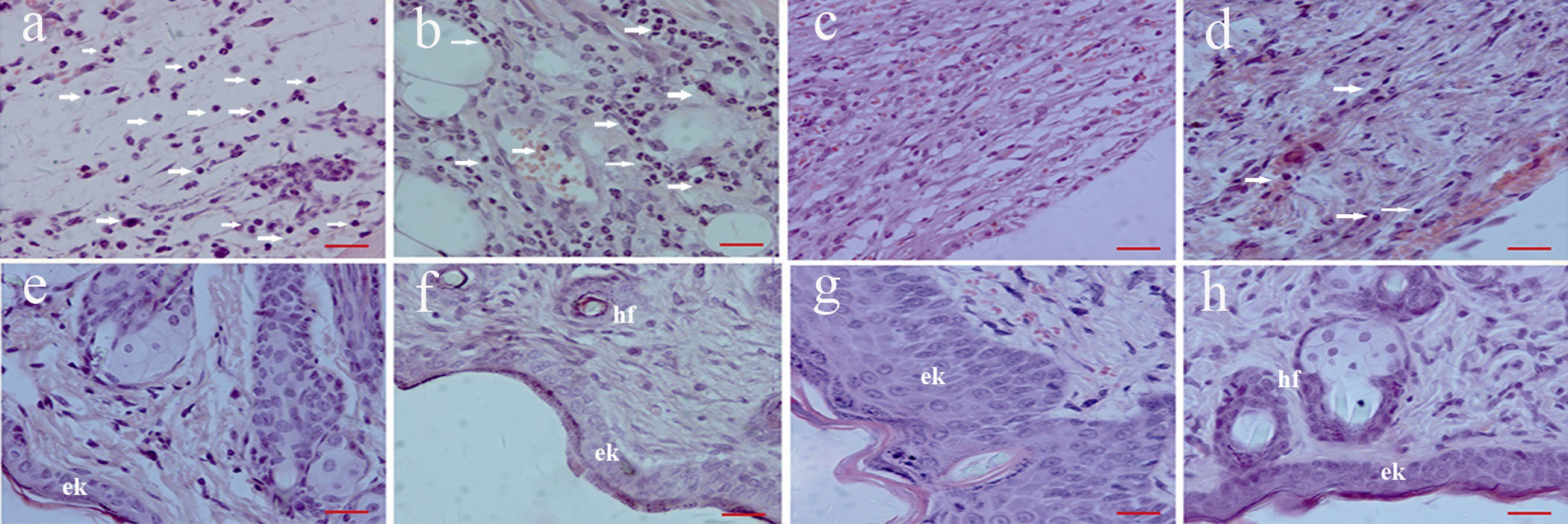
Hematoxylin-eosin–stained sections of mice in the protoporphyrin IX–ethylenediamine derivative-mediated photodynamic antimicrobial chemotherapy (PPIX-ED-PACT) and control groups on days 7 and 14 after treatment. (a, e) Control group on days 7 and 14, respectively; (b, f) 50 μM PPIX-ED-PACT group on days 7 and 14, respectively; (c, g) 100 μM PPIX-ED-PACT group on days 7 and 14, respectively; and (d, h) 200 μM PPIX-ED-PACT group on days 7 and 14, respectively. Images were taken at ×400. Scale bars, 20 μM. White arrows: inflammatory cells. (e, k) Epidermal keratinocytes and (h, f) hair follicles.

Cytotoxicity was examined in the treated and control groups via biopsy on days 7 and 14. On day 7, a lymphocytic infiltrate that extended deeply into the underlying muscle in some sections of the untreated mice was highly prominent. In addition, inflammatory cell infiltration was observed between the dermal adipocytes at the center of the wounds. Moreover, moderate or even heavy bacterial deposits were identified in some wounds, and they were generally confined to areas exhibiting a large fibrin sloughs. Furthermore, a similar inflammatory cell infiltration pattern was identified in the 50 μM and 200 μM PPIX-ED-PACT groups. Conversely, the 100 μM PPIX-ED-PACT group displayed slight tissue necrosis, and the skin was relatively dry with less lymphocytic infiltration. On day 14 after treatment with 100 μM PPIX-ED, considerable newly formed epidermal keratinocytes, newborn hair follicles, and other skin appendages were obviously identified (Fig 10). All specimens exhibited a clear distinction between the wound and the skin, and this finding extended into adipose or loose areolar tissue at their deepest point. However, the control wounds exhibited considerable amounts of disordered slender spindle fibers and amount amounts of interstitial edema cells and collagen fibers.

## Discussion

In this study, the newly synthesized PPIX-ED was identified as an effective photosensitizer exhibiting appropriate photophysical properties (e.g., maximal absorption at a long wavelength, high quantum efficiency, and low toxicity). In addition, it is easy to synthesize, and it achieves a high yield of ^1^O_2_. The present study demonstrated the utility of PPIX-ED as a photosensitizer in the photoinactivation of *P*. *aeruginosa* strains *in vitro* and *in vivo*. Polyamines, which are required for cell development, can increase the biocompatibility and activity of PPIX [43]. Their good biocompatibility resulted in high photoinactivating activity for PPIX-ED against *P*. *aeruginosa*. Our *in vitro* experiments demonstrated that the viability of the bacterium was strongly and concentration-dependently reduced by PPIX-ED-PACT, and PPIX-ED was easily accumulated by *P*. *aeruginosa*.

In the present study, irradiation alone had no effect on the growth of *P*. *aeruginosa*. Moreover, PPIX-ED-PACT (25 μM) eliminated more than 99% of *P*. *aeruginosa* specimens, which was significantly higher than that in the PPIX group. This indicates that polyamines, which are mainly employed as polar groups, increase the uptake of the photosensitizer and thus enhance its combined effect with PACT. These findings are consistent with the hypothesis that gram-negative bacteria are susceptible to cationic photosensitizers [54]. An ideal photosensitizer must absorb light at a compatible wavelength and display high quantum efficiency and low toxicity [55]. The toxicity of the photosensitizer was evaluated following the incubation of NIH3T3 cells with PPIX-ED for 30 min, the same exposure time used in PACT. PPIX-ED displayed relatively high phototoxicity in *P*. *aeruginosa*, whereas it was less phototoxic in NIH3T3 cells. Moreover, PPIX-ED exhibited less toxicity in the dark in both *P*. *aeruginosa* and NIH3T3 cells at concentrations of 3.13–25.0 μM. Confocal laser-scanning microscopy revealed that PPIX-ED was easily accumulated by *P*. *aeruginosa*, but not by NIH3T3 cells, within 30 min. It is suggested that PPIX-ED is more selective for *P*. *aeruginosa* than for NIH3T3 cells, and this selectivity may result from cell envelope differences between the bacteria and fibroblasts. PPIX-ED exerted strong photoinactivation in *P*. *aeruginosa* at a concentration that was weakly cytotoxic to NIH3T3 cells, which indicates that the photosensitizer can be safely used in clinical practice.

Researchers revealed that the roughness, dimensions, and morphology of bacterial strains are distinctly altered after Toluidine Blue O-PACT [56–58]. The leakage of intracellular contents from bacterial strains was higher [56], which suggests that cytoplasmic materials are lost following bacterial membrane damage. In agreement with these findings, the AFM results of this study revealed that PPIX-ED-PACT damaged the bacterial membrane and altered its permeability. This finding was confirmed was using dual fluorescent staining with AO/EB, which may explain the strong antimicrobial effect of PPIX-ED-PACT against *P*. *aeruginosa*.

Our *in vivo* experiments demonstrated that the efficacy of PPIX-ED-PACT against *P*. *aeruginosa* infection in wounds is potent and concentration-dependent. Fourteen days after treatment, the CFUs of *P*. *aeruginosa* in the wound were reduced by factors of 4.2, 3.2, and 3.0 log_10_ at PPIX-ED concentrations of 100, 50, and 200 μM, respectively, compared with 2.6 log_10_ in the control group. This finding was better than that observed for PACT with methylene blue, which decreased bacterial CFUs by a factor of 2 log_10_ in a model of burn wounds infected with *P*. *aeruginosa* [41].

On day 14 after treatment, the wound healing rate was higher in the 100 μM PPIX-ED-PACT group than in the 50 and 200 μM PPIX-ED-PACT groups. Moreover, the average wound healing rate in the 100 μM PPIX-ED-PACT group reached 97.6 ± 2.0%, versus 87.9 ± 3.9% in the untreated control group. The wound healing rate decreased in the order of 100 μM > 200 μM > 50 μM, which parallels the order of the bactericidal effect. This suggests that PPIX-ED-PACT accelerates wound healing via bactericidal effects against *P*. *aeruginosa* [22, 27, 33]. The CFUs of *P*. *aeruginosa* in the wound and the wound healing rate *in vivo* revealed that PPIX-ED-PACT is efficient in the treatment of bacterial infection, and the therapeutic effect of PPIX-ED was better at 100 μM than at 50 or 200 μM.

The clinical value of PACT as a topical antibacterial drug treatment depends on its bactericidal activity and cytotoxicity in host tissues [59]. When the photosensitizer concentration is low, its bactericidal effect is weak, resulting in a low wound healing rate. When the photosensitizer concentration is high, the cytotoxicity of PPIX-ED-PACT in host tissues must be considered. PPIX-ED-PACT can both inactivate bacteria and destroy host tissues, leading to a relatively low wound healing rate. However, at a photosensitizer concentration of 100 μM, the antibacterial effect was optimal, and the cytotoxicity in the host tissue was low, resulting in a superior wound healing rate. These findings reflect the balanced effects of PPIX-ED-PACT on bacterial strains and host tissues. It was further demonstrated using pathological sections that the 100 μM PACT group had the fastest growth rate of granulation tissue, and the rapid proliferation of epithelial cells, endothelial cells, and fibroblasts increased the speed of epithelial crawling, promoted the growth of new capillaries, and facilitated superior wound healing compared with the findings in the other treatment groups and the control group.

## Conclusions

PPIX-ED was synthesized by conjugating an ethylenediamine derivative to PPIX, thereby producing a compound with high photostability, high ^1^O_2_ yields, and selective uptake by bacteria. *In vitro* and *in vivo* experiments revealed that PPIX-ED has potential as an antimicrobial photosensitizer.

## Supporting information

S1 Fig Synthesis of PPIX-2ED. Reagent and reaction conditions: (i) ethyl chloroformate, Et3N, THF, rt, 2 h; ethylenediamine, rt,12 h.
(DOCX)
