# Supplementary material for: Photodynamic antimicrobial chemotherapy in mice with Pseudomonas aeruginosa-infected wounds
Source: PLoS One. 2020 Sep 2;15(9):e0237851. doi: 10.1371/journal.pone.0237851 (PMC7467278; doi:10.1371/journal.pone.0237851)
Supplement: S1 Fig — Reagent and reaction conditions: (i) ethyl chloroformate, Et3N, THF, rt, 2 h; ethylenediamine, rt,12 h. (DOCX) [file pone.0237851.s001.docx]

*Synthesis and Characterization of Photosensitizer*





**Scheme 1.** Synthesis of PPIX-ED. Reagent and reaction conditions: (i) ethyl chloroformate , E_t3_N, THF, rt, 2h; ethylenediamine, rt,12h.

All solvents were analytical grade and used without further purification. Proton nuclear magnetic resonance (^1^H NMR) data were recorded using a Bruker Avance at 300 MHz with d_6_-DMSO as solvent and TMS as an internal standard. High-resolution mass spectra (HRMS) were measured using an Agilent Technologies Agilent 6520 Q TOF series, and infrared spectra were acquired using a Bruker Tensor 27 spectrometer.

Triethylamine (73 µL, 0.534 mmol) was added to a suspension of protoporphyrin IX (0.1 g, 0.178 mmol) in dry tetrahydrofuran (20 mL) at 0 °C, 57 µL (0.534 mmol) of ethyl chloroformate was added dropwise, and the reaction was stirred for 2 h. The solution was then filtered, and excess ethylenediamine (48 µL, 0.712 mmol) was added to the filtrate. The mixture was stirred at room temperature for 12 h, the solvent was removed by evaporation in vacuo, and the crude product obtained was fractionated by passing it through a silica column that was eluted with chloroform–methanol (5:1 (v/v)). The properties of the final product (brown powder, 86 mg, yield 75%) are as follows: TLC (chloroform/methanol, 5:1), R_f_ = 0.2; IR, ν cm^-1^, 3310 (NH), 1636 (-NHC=O); UV-Vis (MeOH), λ_max_ 400, 504, 538, 574, 628 nm; 1H NMR (300 MHz, CD3OD) δ9.44 – 9.01 (m, 4H, meso-H), 8.12 – 7.84 (m, 2H, -CH=CH2), 6.26 –5.96 (m, 4H, -CH=CH2), 3.98 – 3.77 (m, 8H, -CH2CH2CONH-),3.69 – 3.34 (s, 12H, each, 3, 7, 12, 17, methyl), 2.95–2.68 (m, 8H, NH2CH2CH2NHCO-); and HRMS (ESI), m/zcalcd. for C38H47N8O2[M+H]+, 647.3816; found 647.3816.

The protoporphyrin IX was purchased from Aladdin China, The purity of the final product was 98% tested by HPLC normalization method.
